# Hydrogel and Effects of Crosslinking Agent on Cellulose-Based Hydrogels: A Review

**DOI:** 10.3390/gels8090568

**Published:** 2022-09-07

**Authors:** Halimatuddahliana Nasution, Hamidah Harahap, Nisaul F. Dalimunthe, M. Hendra S. Ginting, Mariatti Jaafar, Orlando O. H. Tan, Hotmauli K. Aruan, Alief L. Herfananda

**Affiliations:** 1 Department of Chemical Engineering, Faculty of Engineering, Universitas Sumatera Utara, Medan 20155, Indonesia; 2 School of Materials and Mineral Resources Engineering, Universiti Sains Malaysia, Penang 14300, Malaysia

**Keywords:** natural-based hydrogel, cellulose-based hydrogel, CMCNa-based hydrogel, synthetic crosslinking agent, natural crosslinking agent

## Abstract

Hydrogels are hydrophilic polymer materials that can swell but are insoluble in water. Hydrogels can be synthesized with synthetic or natural polymers, but natural polymers are preferred because they are similar to natural tissues, which can absorb a high water content, are biocompatible, and are biodegradable. The three-dimensional structure of the hydrogel affects its water insolubility and ability to maintain its shape. Cellulose hydrogels are preferred over other polymers because they are highly biocompatible, easily accessible, and affordable. Carboxymethyl cellulose sodium (CMCNa) is an example of a water-soluble cellulose derivative that can be synthesized using natural materials. A crosslinking agent is used to strengthen the properties of the hydrogel. Chemical crosslinking agent is used more often than physical crosslinking agent. In this review, article, different types of crosslinking agents are discussed based on synthetic and natural crosslinking agents. Hydrogels that utilize synthetic crosslinking agent have advantages, such as adjustable mechanical properties and easy control of the chemical composition. However, hydrogels that use natural crosslinking agent have better biocompatibility and less latent toxic effect.

## 1. Introduction

Hydrogels are interlinked hydrophilic polymers that are insoluble in water but are capable of absorbing large amounts of water through the swelling process. During the swelling process, the polar groups in the polymer chain swiftly draw the first water molecules into the hydrogel network (bound water), and the hydrogel network absorbs more water molecules as a result of the osmotic pressure of the interstitial water and free water [1]. Both synthetic and organic polymers can be used to create hydrogels. Natural polymer-based hydrogels are typically chosen because of their excellent biocompatibility and biodegradability. In addition, natural polymers are less expensive than synthetic ones.

With its many benefits, such as biodegradability, good material strength, and environmental friendliness, cellulose, which is a renewable resource found in crops such as straw, maize cobs, bagasse, and water hyacinth, has recently been used to create hydrogels on a large scale and at a low cost [2,3]. However, its use is restricted because it involves a difficult but necessary dissolving procedure. Utilizing chemical processes to transform cellulose into specific derivatives is one way to increase the applicability of the substance [4].

Crosslinked hydrophilic polymer structures called hydrogels have a high capacity for absorbing water and other biological fluids. Chemical linkage of polymer chains with an added crosslink agent affects the physical properties of the polymer depending on the degree of crosslinking and the crystallinity. If the amount of crosslinking agent used is too small, then the physical interaction between polymer bonds breaks easily, causing the hydrogel to become water soluble. However, if too much crosslinking agent is used, then a high crosslinking degree causes a low swelling degree of hydrogel. Crosslinking can make polymer elastic, reduce its viscosity, increase the thermal stability, increase the strength and toughness, lower the melting point (for crystalline polymer with a low degree of crosslinking), and transform thermoplastics into thermosets [5].

Hydrogel can be formed using a variety of crosslinking agents depending on the cellulose derivative that is being used. The most often utilized crosslinking agents for cellulose are epichlorohydrin (ECH) [6], aldehyde-based reagents [7], urea derivatives [8], and multifunctional carboxylic acids [9]. Aldehydes are poisonous when left unreacted [10]. The synthesis of hydrogels made from cellulose that have been crosslinked with citric acid results in hydrogels that are completely safe during the production process and have good swelling capabilities and biodegradability [11].

A wide range of industries, including applied research, healthcare, and agriculture, can benefit from the multifunctionality of hydrogels as a biomaterial. The amazing properties of hydrogels make it suitable for a variety of applications, including the removal of radioactive waste [12], the removal of methylene blue dye from wastewater [13], the engineering of organ tissue [14], the healing of wounds [15], and plant growth media [16]. To prevent its use from later causing additional environmental issues, widely used hydrogels must be backed by sustainable and ecologically beneficial qualities. Researchers are now paying more attention to the synthesis of cellulose-based hydrogels crosslinked with citric acid because these hydrogels satisfy the two criteria.

A significant number of studies and research on a variety of subjects pertaining to hydrogels have been published, according to the literature review. A few review publications have concentrated on cellulose-based hydrogels with different crosslinking agents. This review article focuses on the impact of various crosslinking agents on cellulose-based hydrogels. This review article describes and compares various types of hydrogels based on synthetic and natural polymer derivatives. Hydrogels based on derivatives of cellulose, and types of crosslinking agents for cellulose-based hydrogels, are also discussed in the article. It also includes a thorough discussion of the effects of adding various crosslinking agents to cellulose-based hydrogels.

## 2. Materials and Methods

For this review and viewpoint, information on the several types of cellulose-based hydrogels, natural polymer-based hydrogels, and cellulose derivative-based hydrogels was sought out and gathered. We used two major search engines: Scopus and ScienceDirect. The chosen publications were assessed, evaluated, and interpreted by the writers. The authors’ viewpoint is reflected with regard to the impact of citric acid addition on the characteristics of cellulose-based hydrogels.

## 3. Types of Hydrogels

**Figure 1:**
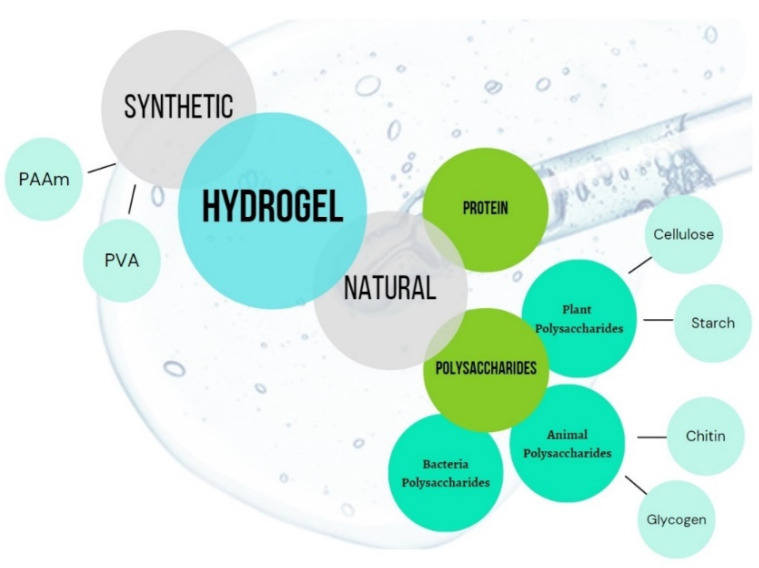
Classification of materials used in hydrogel synthesis.

Zhao et al. [17] reported that hydrogels can be classified into two categories based on their sources: synthetic polymer-based hydrogels and natural polymer-based hydrogels (Figure 1). Earlier research identified two types of synthetic hydrogels: those made from polyacrylamide (PAAm) and those made from poly(vinyl alcohol) (PVA). Polysaccharides produced from plant, animal, and bacterial sources and protein can be used to classify natural hydrogels according to their source. However, the focus of this review article is more on natural polymers of polysaccharides. Cellulose and starch are examples of plant polysaccharide-based hydrogels, while chitin and glycogen are examples of animal polysaccharide-based hydrogels.

### 3.1. Synthetic Polymer-Based Hydrogels

Synthetic polymers are referred to as human-made polymers. The advantages of hydrogels made from synthetic polymers include facile chemical composition control and changeable mechanical qualities. However, compared with hydrogels based on synthetic polymers, hydrogels based on natural polymers have greater biocompatibility and less latent toxic effect [18]. They are therefore more inert than hydrogels made from organic biomaterials. Synthetic hydrogels are easier to adapt, have a larger water retention capacity, and have a longer shelf life than natural hydrogels. These benefits have led to the use of synthetic polymers as scaffolding for various cell cultures, including stem cells, to enhance tissue engineering [19]. Table 1 describes the types of synthetic polymers made from PAAm and PVA, the types of crosslinking agents used, and the results.

The data in Table 1 evidently show that Maijan et al. [20] successfully created PAAm-based hydrogels by using N,N′-methylenebisacrylamide (NMBA) as a crosslinking agent. The initial concentration of the methylene blue (MB) solution was 50 ppm, the adsorbent dose was 0.5 g/100 mL, and the contact time was 3 h. The hydrogel created with PAAm was able to absorb almost 90% of the MB dye. However, this can be accomplished by adding natural rubber (NR) to a hydrogel to result in natural rubber-graft-polyacrylamide (NR-g-PAAm). The formation of the semi-IPN structure with NR chains improved the mechanical properties while maintaining the adsorption performances of the hydrogels. Hydrogels without the addition of NR have a lower water absorption than hydrogels with the addition of NR. Pure NR (100 NR) did not swell due to the hydrophobic nature of NR. The high level of free NR restricted chemical crosslinks between polymer chains, leading to the formation of very large pores. The hydrogel with large pores could not retain a large amount of water, and water absorption was low. Therefore, the high NR loadings slightly reduced the mechanical stability of the hydrogels.

These results suggested that the mechanical and adsorption performance of the hydrogel was critically affected not only by the presence of the secondary network in the semi-IPN but also the porous structure of the hydrogel.

In line with previous research, Garcia et al. [21] found that the swelling capacity of NMBA-free PAAm-CA (cellulose acetate) hydrogels is comparable to that of formulations with NMBA for CA concentrations higher than 20 wt%. This condition occurred because CA crosslinks PAAm through free radical reactions. The hydrogel obtained without crosslinking NMBA is suitable for tissue engineering applications such as cartilage replacement, as it exhibits a compression modulus of up to 1.7 MPa.

In line with previous research, Muhamad et al. [22] synthesized PAAm, PAAm/CMCNa, and PAAm/CMCNa/magnesium oxide (MgO)-based hydrogel using NMBA as a crosslinking agent, ammonium persulfate (APS), and N,N,N′,N′-tetra methylethylenediamine (TEMED) as an initiator. The quantity of edema (the medical term for swelling) was significantly increased by the addition of CMCNa. The structure of CMCNa contains many carboxylic acid groups. It is a smart cellulose derivative polyelectrolyte that exhibits excellent swelling properties, pH sensitivity, and ionic strength fluctuations. Increased hydrogel swelling may result from the presence of more hydrophilic chains or from the hydration of functional (–OH and –CH_2_COONa) groups on polymeric chains. The swelling capacity, on the other hand, is adversely affected by MgO nanoparticles. Lowering the porosity of the gel can enable these nanoparticles to alter the hydrogel network’s structure. Therefore, the network becomes tougher and the hydrogel’s ability to swell is inhibited by the MgO nanoparticles’ presence.

Meanwhile, Wu et al. [23] successfully created hydrogels based on PVA and other synthetic polymers by using ECH as a crosslinking agent. Additional materials, mainly lignin, should be incorporated in hydrogels to boost their adsorption capacity due to the characteristics of PVA-based hydrogels, which have a low adsorption capacity and mechanical qualities.

In line with previous research, another PVA-based hydrogel was synthesized by Mazzuca et al. [24] with telechelic PVA as a crosslinking agent. Telechelic PVA is PVA bearing an aldehyde at each chain end. The results show a higher amount of telechelic PVA as a crosslinking agent than PVA, as polymers can reduce the total water content (TWC). Gel can retain a significant moisture content for use in cleaning paper artwork. Therefore, having a high TWC value means that the hydrogel can be used for cleaning paper artwork.

In line with previous research, Djumaev and Tashmukhamedova [25] synthesized PVA/CMCNa hydrogel by utilizing inebrin as a hemostatic agent. Up to a specific swelling limit, the hydrogel’s maximal absorption capacity improved with increasing CMCNa content. In the absence of CMCNa, a structure with a high degree of crosslinking was obtained; however, this structure was not able to store a large amount of water, thereby limiting the ability to swell, which was reduced to roughly 500%. The percentage of water absorption steadily rose to roughly 3200% after the CMCNa content was increased because a high CMCNa level enhances the hydrophilicity of the hydrogel, thereby occasionally resulting in the partial or total disintegration of hydrogel with a much higher CMCNa content.

However, hydrogels based on natural polymers are known to have greater biocompability and less latent toxic effect than hydrogels based on synthetic polymers [18]. This condition is due to the fact that hydrogels made from natural polymers, particularly those derived from polysaccharides, are comparable to living natural tissues that can absorb high water content and are biocompatible and biodegradable [26,27].

### 3.2. Natural Polymer-Based Hydrogels

Natural hydrogels are frequently used for stem cell control and culture, because they offer highly desirable qualities, including biocompatibility and biodegradability. Natural hydrogels contain a variety of intricate components that can improve cellular performance and encourage the proliferation, viability, and diversification of different cell types [18]. The types of hydrogels made from natural polymers based on polysaccharides from plant, animal, and bacterial sources are explained in the following subsection.

#### 3.2.1. Plant Polysaccharide-Based Hydrogels

Many plant polysaccharide-based hydrogels have been developed to date. Table 2 summarizes several types of plant polysaccharide-based hydrogels; the types of natural polymers, whether cellulose or starch; the types of crosslinking agents used; and the results. 

On the basis of previous research reported in Table 2, Golor et al. [28] synthesized sugarcane bagasse cellulose-based hydrogel, with citric acid and ECH as a crosslinking agent. It was found that the amount of citric acid that was added to sugarcane bagasse cellulose affected the hydrogel formation and the degree of its friability. The addition of citric acid in low amounts did not accommodate the formation of crosslinked networks; as a result, the hydrogel could not be formed and became brittle. With a smaller amount of ECH, hydrogels could be formed. This condition indicates that citric acid is a weaker crosslinking agent than ECH. This weaker crosslinking is due to its acidic characteristics, which can undeniably interfere with the dissolution of cellulose. However, despite the requirement of a higher amount of citric acid in the hydrogel preparation, citric acid is more environmentally friendly than ECH, being non-carcinogenic and non-toxic. 

In line with previous research, Enawgaw et al. [29] synthesized a corncob cellulose-co-AMPS (2-acrylamide-2-methylpropane sulfonic acid)-based hydrogel by using a crosslinking agent in the form of borax decahydrate and potassium persulfate (KPS) as an initiator. It was found that the manufactured hydrogel had a swelling ratio to urine solution lower than that of hydrogel to water. With the presence of other substances in urine besides water, such as urea, sodium chloride, and potassium sulfate, the aqueous solution’s composition changed, thereby leading to the decreased absorption of hydrogel in the urine solution.

In line with previous research, Kadry et al. [30] synthesized hydrogel with cellulose, CMCNa, and CMCNa/cellulose as a polymer, which was grafted with PAA and created hydrogels using vinyl sulfone (VS) as a crosslinking agent and GA, NMBA, and ECH for the 1:1 mixture of cellulose: CMCNa. CMCNa hydrogel, CMCNa: cellulose (1:1) (wt/wt) hydrogel, and CMCNa: cellulose (4:1) (wt/wt) hydrogel all showed high-equilibrium swelling after three days and reached saturation on the fourth day, while cellulose hydrogel reached 7182% on the fourth day. This finding indicates that cellulose-based hydrogel has better absorption properties that are less restricted than those of other hydrogels. Additionally, this condition may be because of its alkalinity and the presence of –OH group in the chain, thereby decreasing the crystallinity and increases the swelling of the cellulose chains. Increasing the ratio of CMCNa compared with CMCNa/cellulose increased the rate of absorption until the third day, and on the fourth day, it reached saturation. This finding indicates a correlation between the presence of cellulose and CMCNa and the saturation state, where the acrylic acid (AA) changes to sodium acrylate and CMCNa has a high hydrophilic carboxylic group (COO–) with high electronegativity and polarity that can physically interact with the water molecules. Therefore, the electrostatic repulsion of carboxylate groups can expand the polymer chains and lead to greater water adsorption. The 1:1 CMCNa: cellulose hydrogel was created using VS, glutaraldehyde (GA), ECH, and NMBA as crosslinking agents. The GA crosslinking agent produced the highest absorption rate among all crosslinking agents.

The best features of hydrogels with a satisfactory swelling ratio can be achieved by synthesizing hydrogels made of cassava starch with the use of ECH and functionalized carboxymethyl crosslinker (SEC) as a crosslinking agent. However, the swelling ratio of hydrogel decreases with the addition of more than 5–10% ECH and SEC. To shorten the gap between the crosslink chains, the number and size of the pores must be decreased. The hydrogel’s water absorption capacity decreases with decreasing number of pores, because a small surface area of the hydrogel is in contact with water [31].

In line with previous research, Nicolic et al. [32] synthesized starch-based hydrogel using citric acid as a crosslinking agent. It was found that the maximum swelling degree of the hydrogel was achieved by placing it in water with pH = 7 with a 72 ratio of glucose units of starch and citric acid. The higher starch-to-citric acid ratios did not produce any hydrogel in the process.

In line with previous research, Halim and Deyab [33] synthesized hydrogel with 10 g of poly(acrylic acid)/starch graft copolymer (PASGC) as a polymer and used 1–10 g of ECH as a crosslinking agent for their research. It was found that with an amount of up to 5 g of ECH, the ability of the hydrogel to swell increased with increasing amount of ECH. Thus, it makes sense that raising the amount of ECH would boost the swelling capacity because it strengthens the network structure of the hydrogel by adding more crosslinks to the hydrogel. Moreover, when the amount of ECH exceeded 5 g, the swelling capacity of the hydrogel synthesized decreased continuously. When the amount of ECH exceeded a particular threshold, the hydrogel network structure exhibited a higher crosslinking density, which could explain the decrease in swelling capacity. The network structure of the hydrogel cannot absorb more water molecules due to the high crosslinking density of the hydrogel.

#### 3.2.2. Animal Polysaccharide-Based Hydrogels

The literature indicates that many animal polysaccharide-based hydrogels have been developed. Table 3 summarizes the types of animal polysaccharide-based hydrogels reported in previous research and describes the types of polymer (normally chitin and glycogen), the types of crosslinking agents used, and the results. 

Table 3 shows that Liao et al. [34] successfully synthesized a chitin-based hydrogel by using the residue of the fungus *Hericium erinaceus*, converting it to carboxymethyl chitin, and making hydrogels with ECH as a crosslinking agent. Different concentrations of sodium monochloroacetate (MCA) were added to the chitin solution to prepare carboxymethyl chitin with different degrees of substitution (DS). The highest DS of 0.038 was obtained with the highest MCA concentration of 0.7 g/mL, indicating that the DS of carboxymethyl chitin increases with increasing MCA. The highest equilibrium swelling degree of 40.2 g/g was obtained with the highest DS value of 0.038. To further compare the swelling capacity of hydrogels, the diameter and equilibrium swelling degree of the hydrogels at final stage were measured. The diameter of the prepared hydrogels was 2.40 cm in the initial stage, but after swelling, it increased to 3.51 cm, with a growth rate of 46.3% for the hydrogel, with a maximum equilibrium swelling degree of 40.2 g/g. These results further prove that the DS has a positive effect on the swelling ability of hydrogels; as DS increases, the swelling ability gradually increases.

In line with previous research, Huang et al. [35] synthesized regenerated chitin nanofiber hybrid (RCNs)-poly (ethylene glycol diglycidyl ether) (PEGDE) as the first network and PAAm as the second network (RCNs-PEGDE/PAAm) hydrogel with NMBA as crosslinking agent. Chitin is a biopolymer that is used for biomedical materials, yet their weak mechanical properties limit their potential. Unlike native chitin nanofibers, the RCNs-based hydrogels can hold a large amount of water. However, they are weak and brittle. Double-network (DN) strategies, such as PAAm, can efficiently overcome mechanical weaknesses. A DN hydrogel consists of two interpenetrated networks with contrasting structures: the first network is rigid and brittle, and the second network is soft and stretchable. Moreover, the swelling capacity of DN is greater than that of SN hydrogel.

In line with previous research, He et al. [36] used ECH as a crosslinking agent to create a chitin–PVA hydrogel. The equilibrium swelling ratio for chitin was steadily reduced as the PVA content increased, showing that the structure of the gels with PVA was denser than that of the gels made entirely of chitin. Therefore, varying the chitin/PVA ratio for diverse purposes can help in the creation of hydrogels with variable pore diameters and equilibrium swelling ratio values between 11.8 and 52.5.

Another natural polymer used for hydrogel synthesis is glycogen. A self-healing hydrogel was developed by Hussain et al. [37] by inserting Fe^3+^ ions as a crosslinking agent between glycogen–PVA and PAA in the presence of APS via the sol–gel method. The prepared hydrogels consisted of a triple-network system and possessed two types of non-covalent interactions within the hydrogel network. The –OH groups on the glycogen formed hydrogen bonding interactions with the functional groups of PVA and PAA, while the carboxylic groups of PAA chain and the –OH groups on glycogen and PVA formed ionic coordination interactions with the metal Fe^3+^ ions. It was shown that it had outstanding self-healing efficiency, stretchability, and customizable mechanical properties. 

In line with previous research, Hussain et al. [38] synthesized hydrogel with commercial glycogen, PAA and PAAm as a polymer and used iron (III) as a crosslinking agent. It was found that, with the addition of commercial glycogen, the tensile stress and strain of the hydrogel synthesized were high. However, at higher glycogen concentrations, the tissue became hard and brittle, thereby decreasing its tensile strength. However, because metal ions may move freely and natural polymer chains are dynamic, the hydrogel can demonstrate strong ionic conductivity, making it a more effective biomaterial for conducting electricity and for self-healing.

In line with previous research, Patra et al. [39] synthesized glycogen and N-isopropylacrylamide (NIPAm) hydrogel using ethylene glycol dimethacrylate (EGDMA) as a crosslinking agent and KPS as an initiator through conventional free radical polymerization. Elastic modulus (G′) and loss modulus (G″) declined steadily, and after a specific shear stress, they underwent a sharp reduction, indicating the breakup of hydrogel. The shear stress is called the yield stress (σ) of the hydrogel. With an increase in the frequency, a typical rising trend in yield stress was seen (0.1–10 Hz). This condition can be explained by the fact that when the frequency rises, the polymer chains vibrate fast and are unable to rearrange themselves to conform to the forced motion, producing a stiff polymeric network. As a result, the polymer chains behave more rigidly, are solid-like, and have a higher gel strength at higher frequencies than at lower frequencies. Therefore, higher-frequency shear stress is needed to break the hydrogel by using a lower-frequency shear stress. In acidic medium (pH 1.2), the equilibrium swelling ratio is smaller than in basic medium (pH 7.4). This effect can be explained because hydrophilic groups in the hydrogel network become protonated in acidic buffers, preventing the formation of H-bonds with water molecules and leading to a decreased swelling ratio. In alkaline conditions, the hydrophilic moieties are kept in an unprotonated state, which encourages the formation of H-bonds with water molecules and results in a larger swelling ratio. The swelling ratio of the hydrogel also decreased with increasing temperature. At 25 °C below of lower critical solution temperature (LCST) the hydrophilic groups of the polymer molecule interact with the water molecules through intermolecular H-bonding, resulting higher swelling ratio. When the temperature was raised to 37 °C above of LCST, water molecules gain a certain amount of enthalpy and the hydrophilic groups of the hydrogel network form intramolecular H-bonding, so hydrophobic force predominates over hydrophilic force, resulting in lower swelling ratio.

#### 3.2.3. Bacteria Polysaccharide-Based Hydrogels

According to the literature, some bacteria polysaccharide-based hydrogels have been developed. Table 4 summarizes the types of bacteria polysaccharide-based hydrogels reported in previous research, the types of polymer, the types of crosslinking agents used, and the results.

Table 4 shows that Siripongpreda et al. [40] successfully in synthesizing a bacterial cellulose (BC)/CMCNa-based hydrogel. The BC/CMCNa hydrogel was re-swellable and could be made without requiring special physical or chemical treatment, but with the direct deposition of the negatively charged polyelectrolyte, CMCNa, into the BC matrix. The BC/CMCNa-based colorimetric pH sensor exhibited a rapid response with an easy color differentiation between each pH by the naked eye and a wide linear range of pH 4.0–9.0 with good linearity. The colorimetric glucose sensor was based on the color development of KI by hydrogen peroxide (H_2_O_2_) from the enzymatic reaction of glucose oxidase enzyme (GOx). Briefly, 10.0 μL of 0.5 M KI was dropped onto the BC/CMC hydrogel, followed by 2.5 μL of the enzyme mixture to generate the BC/CMC-based glucose sensor. This hydrogel has the potential to be a platform for non-invasive sensors for sweat and glucose pH due to its high water absorption capacity, which is advantageous for effective collection of biofluid samples and provides high analytical performance, including wide linear detection and low detection limits with low sample volume requirements. 

In line with previous research, Treesuppharat et al. [41] synthesized a BC and gelatin-based hydrogel with GA as a crosslinking agent. The hydrogel was successfully prepared with GA as a crosslinking agent between the hydroxyl group of bacterial cellulose and the amine group of gelatin. Gelatin that was inserted into the cavity of the bacterial cellulose network showed good compatibility with bacterial cellulose. Hydrogel composites provided the benefits of thermal stability, chemical resistance, and good mechanical properties.

In line with previous research, Khattak et al. [42] synthesized a BC and chitosan (CS)-based hydrogel with GA as a crosslinking agent. It was found that BC-CS hydrogels incorporating SSd could be prepared with GA as a crosslinking agent.

**Figure 2:**
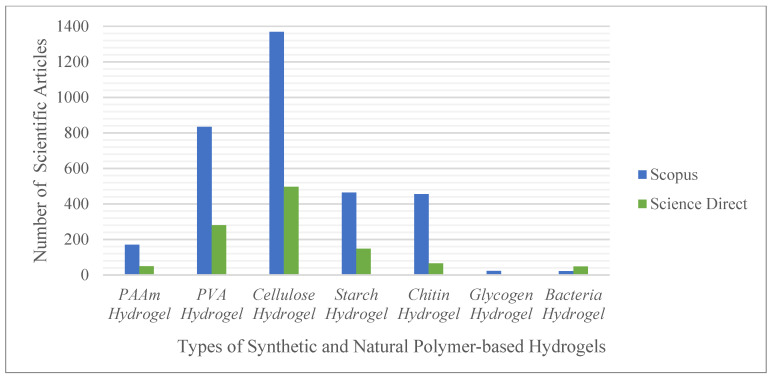
Statistics of the search results on the scientific search engines Scopus and ScienceDirect.

Based on the explanation of the previous articles, cellulose is the most popular natural polymer used in the synthesis of hydrogels. This statement is based on the statistical data search results on the scientific search engines Scopus and ScienceDirect concerning the four natural polysaccharide polymers, namely, cellulose, starch, chitin, and glycogen. The following keywords are used: “PAAm-based hydrogel”, “PVA-based hydrogel”, “cellulose-based hydrogel”, “starch-based hydrogel”, “chitin-based hydrogel”, “glycogen-based hydrogel” and “bacteria-based hydrogel”, as shown in Figure 2. 

Many scientific articles related to cellulose-based hydrogels have been published recently (1369 and 496 articles found in Scopus and ScienceDirect, respectively, in 2011–2021). This finding indicates that cellulose is the most popular natural polymer used as raw material. With its abundant availability worldwide and ability to combine hydrophilicity with good mechanical properties, cellulose is being increasingly widely used [27].

In terms of ability, cellulose has a stronger structure, higher degree of crystallinity [27], and very high swelling ratio [29,30] than other polysaccharide polymers such as starch and glycogen for hydrogel materials. This is because starch and glycogen are connected to α-1.4 or α-1.6 glycosidic chains, while cellulose is connected to β-1.4 glycosidic chains, which means that it will form a structure in the form of fibers and be more resistant to degradation because the position of monomer residues in cellulose is reversed (trans configuration) and bonded well with an increasing number of chain bonds. These two things make cellulose a raw material that can potentially be used to produce hydrogels from natural polymers.

## 4. Cellulose-Based Hydrogels

### 4.1. Cellulose

**Figure 3:**
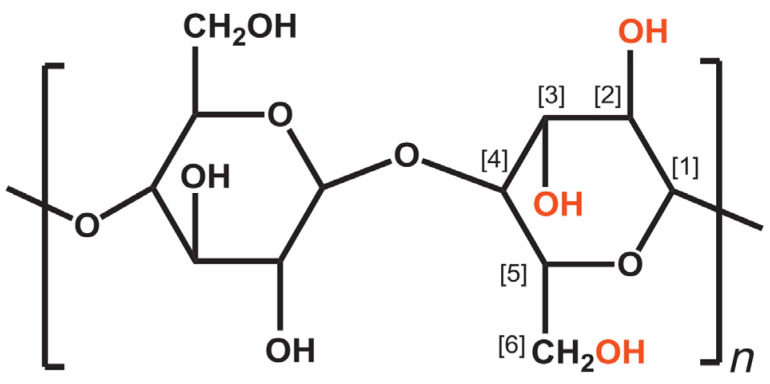
Molecular structure of cellulose (*n* = DP, degree of polymerization) [45].

The fundamental structure of plant cell walls is cellulose, and in certain woods, cellulose accounts for about 40–50% [43]. Cellulose is constructed from glucose chains linked via −1.4 glycosidic bonds formed between C_1_ and C_4_ of adjacent glucose groups. Each D-anhydroglucopyranose has three hydroxyl groups (OH) at positions C_2_, C_3_, and C_6_, as shown in Figure 3 [44].

The OH group on C_1_ is the OH found in aldehydes, referred to as reducing agents. This aldehyde group forms a pyranose ring through an intramolecular hemiacetal form. The OH groups on D-anhydroglucopyranose are one primary OH group and two secondary OH groups. In C_2_ and C_6_–OH groups, intermolecular hydrogen bonds form. In the C_3_–OH group and oxygen on the pyranose ring, intramolecular hydrogen bonds form. Intramolecular and intermolecular hydrogen bonding occurs due to the large number of OH groups in cellulose [46].

The use of cellulose as raw material is preferred in the manufacture of hydrogels based on natural polymers because of its inherent biocompatible and biodegradable properties, in addition to the excellent availability of various types of functional groups that can be used for modification, bio-adhesion, biocompatibility, accessibility, and affordability [15,47].

### 4.2. Synthesis of Cellulose-Based Hydrogels

**Figure 4:**
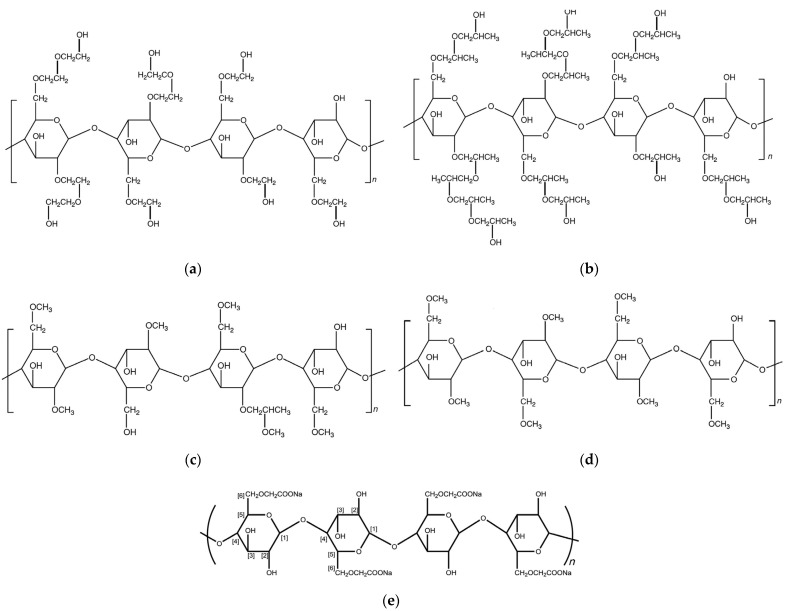
Molecular structure of (**a**) HEC (DS = 1.75); (**b**) HPC (molar substitution [MS] = 4); (**c**) HPMC (hydroxypropyl DS = 0.25 and methoxyl DS = 1.5); (**d**) MC (DS = 1.75); (**e**) CMCNa (DS = 1) [45].

Several cellulose derivatives that have been developed to synthesize hydrogels include methylcellulose (MC) [48], hydroxyethyl cellulose (HEC) [49], hydroxypropyl cellulose (HPC) [3], hydroxypropyl methylcellulose (HPMC) [50], and carboxymethyl cellulose sodium (CMCNa) [51]. These derivatives are known to be water-soluble cellulose derivatives. Figure 4 shows the molecular structure of HEC, HPC, HPMC, MC, and CMCNa. Table 5 summarizes previous research related to the synthesis of cellulose-based hydrogels, the types of cellulose derivatives and crosslinking agents used, and the results. 

MC is a macromolecule of cellulose, with 27–32% of the hydroxyl group in the form of methyl ether. Various grades of MC with degrees of polymerization in the range of 50–1000, molecular weights in the range of 10,000–220,000 Da, and degree of substitution in the 1.64–1.92 range are commercially available [52]. This methyl derivative of cellulose has the special property of forming a thermally reversible hydrogel upon heating, thus being classified as a polymer with a lower critical solution temperature [53].

Bonetti et al. [48] developed MC-based hydrogels with citric acid as a crosslinking agent. In the first 24 h, all hydrogels showed an increase in weight due to water absorption. Swelling balance is reached in the next 24 h. Increasing the degree of crosslinking of the sample causes a significant decrease in the swelling ratio. The equilibrium swelling degree of hydrogels prepared with a constant amount of MC is dependent on the amount of critic acid, with the average swelling values ranging from 800% for MCs with 5% citric acid to 3000% for MCs with 3% citric acid. Conversely, MC with 1% citric acid did not show significant differences in terms of swelling at the equilibrium compared with MC control. This finding indicates that the specimen’s swelling behavior is slightly affected by low crosslinking. In fact, an increase in the crosslinking degree causes an increase in crosslinking points, preventing crosslinked MC network expansion in the water environment.

In line with previous research, Quiroz et al. [54] synthesized MC-based hydrogel with citric acid as a crosslinking agent. Citric acid functions as a crosslinking agent for MC hydrogels when used at low concentrations (5% *w*/*w*). The crosslinking decreased water vapor permeability and swelling, allowing good gas barrier properties to be obtained. The formulation of MC 1.5%, 0.25% sorbitol, and 5% citric acid (*w*/*w* MC) would allow reduced-affinity coating for water and oxygen to be obtained, which can be used to cover foods under low-humidity conditions and preserve nutrients susceptible to oxidation.

HEC is a partially substituted hydroxyethyl etherified cellulose. It is a hydrophilic polymer with a degree of substitution of at least 1.5. When the degree of substitution of HEC increases, the level of solubility in water will increase [53]. With its biocompatibility and non-immunogenicity, HEC is often used as stabilizer, thickener, film, hydrogel, nanofiber in tissue engineering applications, and it can improve the quality of the resulting hydrogel both mechanically and rheologically [55,56,57,58]. 

Fawal et al. [49] developed an HEC-based hydrogel with citric acid as a crosslinking agent and tungsten trioxide (WO_3_) as a support material for wound dressing applications. The FTIR analysis showed the presence of HEC and citric acid and that crosslinking had occurred. The gel fraction of hydrogel without WO_3_ and with 0.02% WO_3_ was 59.7% and 65.9%, respectively. Swelling or the highest water absorption was 300.1% without WO_3_ and 165.6% with 0.02% WO_3_, and decreased with increasing WO_3_. The percent of water absorption decreased with increasing concentration of WO_3_, because WO_3_ consumes some hydrogen bonds.

In line with previous research, Wang et al. [58] synthesized hydroxyethyl cellulose-g-poly(sodium acrylate)/medicinal stone (HEC-g-PNaA/medical stone)-based hydrogel with NMBA as a crosslinking agent. The addition of various amounts of medical stones can change the structure and composition of the hydrogel and affects the swelling capacity. With a medical stone of up to 10% by weight, the swelling capacity increased sharply by 400% and then decreased with further addition of medical stone. The addition of medical stone can decrease the degree of physical crosslinking and increase the swelling capacity because when NaA was grafted onto HEC and MS can participate in the polymerization reaction through its active silanol groups, contributing to the formation of ordinary polymer networks, preventing the intertwining of grafted polymer chains, and weakening hydrogen bonding interactions between groups. However, when the addition of medical stone exceeded 10% by weight, the swelling capacity decreased, because the tissue cavity for holding water was blocked and the hydrophilicity of the hydrogel decreased.

HPMC is a propyleneglycol ether of methylcellulose, described by the PhEur as a partly O-methylated and O-(2-hydroxypropylated) cellulose. HPMC is a water-soluble polymer that is available in several grades with different viscosities and substitution rates. HPMC hydrogel has high levels of transparency, stability, and viscosity because of its good biocompatibility and thermosensitive natural polymers [53,59,60]. 

Seyedlar et al. [50] developed HPMC-based hydrogels with biphasic calcium phosphate (BCP) that were applied to tissue engineering. HPMC-based hydrogels can reduce the invasiveness of osteoplasty surgery, shorten the operating time, and cause homogeneous cell distribution. Incorporation of hydroxyapatite (HAp) and β-tricalcium phosphate (TCP) nanoparticles on BCP in an HPMC aqueous solution increased the viscosity of injection scaffold but decreased the gelation temperature.

In line with previous research, Bashir et al. [61] synthesized HPMC hydrogel with HPMC-pectin-co-acrylic acid as a polymer and NMBA as a crosslinking agent. PAA containing COOH group is the reason for the increase in the swelling pattern, which has a greater tendency to ionize as the high porosity of hydrogel increases at pH 7.4. The HPMC formulation gradually increased from 0.5 g to 1.5 g, causing the percentage of drug release to also increase simultaneously from 75.36% to 87.62% at pH 7.4, because HPMC has higher swellability and hydrophilic properties at pH 7.4.

HPC is a polymer in which some of the hydroxyl groups of cellulose have been hydroxypropylated, forming -OCH_2_CH(OH)CH_3_ groups. During the HPC manufacturing process, the added hydroxypropyl group can be esterified, having a mole substitution value (number of moles of hydroxypropyl groups per glucose ring) greater than 3. Therefore, HPC must have a degree of substitution (DS) value of 2.5 and a molarity of substitution (MS) of 4 to have good water solubility [52,62,63,64].

Chen et al. [3] developed HPC-based hydrogels made by modifying HPC to alkynyl-HPC as a polymer and molybdenum disulfide (MoS_2_) as a crosslinking agent. The hydrogels produced from this study had high water absorption capabilities and thicker pore walls. The addition of MoS_2_ with HPC can make the hydrogel to be effective in removing methylene blue dyes. The addition of MoS_2_ into HPC can induce a reduction in the swelling ratio of the hydrogel because the addition of MoS_2_ into HPC weakens the effect of the volume phase transition of hydroxypropyl cellulose, which causes an increase in crosslinking.

In line with previous research, Yan et al. [65] synthesized HPC hydrogel with ECH as a crosslinking agent, and ammonia as a co-crosslinking agent. It was found that the adsorption ability of the resin had a strong relationship with the pH value. The microporous structure and the chemical structure of the prepared crosslinked HPC resin are the key factors in producing hydrogels with high adsorption capacity of anionic dyes. The resin can also be used in neutral conditions with a high adsorption capacity for anionic dyes.

CMCNa is a hydrophilic polymer prepared by partial substitution of OH groups in the second, third, and sixth positions of cellulose by carboxymethyl groups. The DS value varies in the range of 0.6–1, affecting several physicochemical properties of the polymer. Therefore, due to the higher DS value, the water solubility and sodium content of CMCNa increase and the polymer tolerance for other components in the solution improves [53]. 

Alam et al. [51] developed a CMCNa-based hydrogel with ECH as a crosslinking agent. FTIR analysis showed the presence of CMCNa and ECH, as well as the fact that crosslinking had occurred. The hydrogel with the highest water absorption or water retention value (WRV) was obtained with a composition of 3% of CMCNa and 4% of ECH.

In line with previous research, Astrini et al. [66] synthesized CMCNa hydrogel with divinyl sulfone as a crosslinking agent. The weight loss of CMCNa and crosslinked CMCNa/HEC hydrogels indicated a loss of moisture in the samples when the temperature increased (100–170 °C). The TD was 285.5 °C (68.2% weight loss) for CMCNa and 276.6 °C (56.8% weight loss) for crosslinked CMCNa/HEC (5/1). The peak temperature of the main degradation step of CMCNa/HEC (5/1) shifted to a lower temperature compared with pure CMCNa. The crosslinked structure plays an important role in thermal decomposition and indicates that CMCNa is more stable than CMCNa/HEC. With increasing synthesis temperature and reaction time, water absorption capacity also increased. 

As a polyelectrolyte, CMCNa is sensitive to pH and ionic strength. Therefore, the compatibility of CMCNa in a solution with other components is an important characteristic. CMCNa is highly compatible with most 10% and 50% monovalent inorganic salt solutions of the cations that form CMCNa soluble salts. Crosslinked CMCNa is capable of absorbing large amounts of water and swells to form superabsorbent hydrogels that exhibit superior mechanical and viscoelastic properties compared with other crosslinked cellulose derivatives hydrogels [1].

CMCNa-based hydrogels can be used in enzyme immobilization, wound healing, drug delivery, and adsorbents. They can be made into materials for applications involving anti-bacterial activity, drug delivery, wound healing, and tissue engineering [67,68,69]. CMCNa is easily synthesized from cellulose derived from waste biomass extraction, such as oil palm empty fruit bunches and bagasse because it provides unique CMCNa properties, such as good adsorption, high swelling capacity, and good optical properties (i.e., how it interacts with light, focusing on biomedical applications). The high methylation group in the biomass waste is also an advantage for the production of CMCNa-based hydrogels.

**Figure 5:**
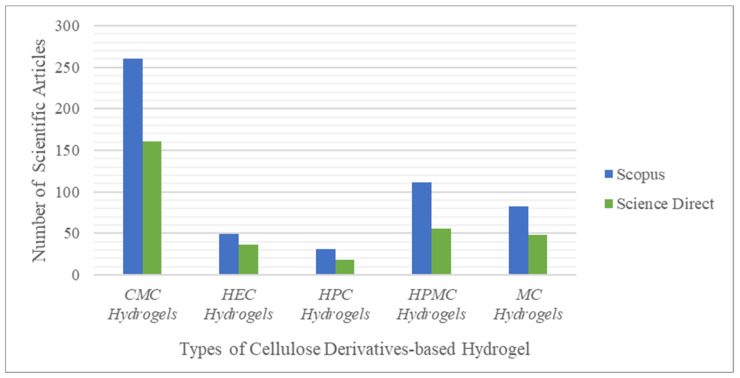
Statistics of the search results for scientific articles on Scopus and ScienceDirect 2.

Among the five cellulose derivatives mentioned above, CMCNa remains a favorite raw material for developing hydrogel materials. This is supported by the statistics shown in Figure 5, obtained from Scopus and ScienceDirect.

The statistical data in Figure 5 were collected by searching for related articles using several keywords, such as “MC hydrogel,” “HPMC hydrogel,” “HEC hydrogels,” “HPC hydrogel,” and “CMCNa hydrogel,” in the years ranging from 2011–2021. The data indicate that Scopus and ScienceDirect had 260 and 161 scientific articles on the topic of CMCNa-based hydrogels, respectively. This result may be due to the nature of CMCNa itself; CMCNa exhibits a relatively constant level of viscosity over a wide temperature range. The carboxyl group present in CMCNa is the reason for this advantage, because the addition of the carboxyl group to cellulose can adjust the properties and allow the end user to obtain a certain texture beyond the thickness. CMCNa also has high water absorption [51] and swelling ratio [30] when used for hydrogel materials.

Most CMCNa that is used as a raw material in hydrogel synthesis is made from natural materials. Research on manufacturing CMCNa with natural ingredients has been conducted in the past. Rachtanapun et al. [70] reported cellulose from durian rind isolated with NaOH and bleached with hydrogen peroxide. The cellulose was converted to CMCNa using various NaOH concentrations for carboxymethylation. The best results showed that the DS values increased with increasing NaOH concentrations.

Recently, Phan and Thi [71] synthesized CMCNa from another natural material, namely, passion fruit peel cellulose. Passion fruit peel has excellent potential with a dry weight of cellulose of about 42% [71] and high cellulose content of about 86.2 g/kg [72]. They conducted an experiment to extract the cellulose from passion fruit peel, which was then synthesized into CMCNa. The highest cellulose extraction yield was 32.13% at 1 M NaOH and 1.25 M HNO_3_. The obtained cellulose was then characterized using FTIR; several peaks were observed, indicating that the cellulose produced was pure cellulose and showing the presence of β-(4, 17)-glycosidic linkages between the glucose units in cellulose. This cellulose was synthesized into CMCNa, with a maximum CMCNa yield of 79.5% and a degree of substitution of 0.78, which were achieved at 20% NaOH concentration and 2 g monochloroacetic acid (MCA). The functional groups of CMCNa were analyzed using FTIR. The presence of –COO and –COONa groups was observed, indicating that cellulose etherification was successful. 

Many studies have been conducted on the manufacture of CMCNa from various natural materials, with good and high yields; therefore, CMCNa from natural materials has the potential to be used as a raw material in the manufacture of hydrogels. In particular, passion fruit peel has been used only as a feed mixture [73] and in the manufacture of pectin extracts [64]. In the material sector, passion fruit peel is only used as a film [74], activated carbon [75], and microcrystalline cellulose [76].

In recent decades, crosslinked CMCNa networks have been obtained by applying crosslinking technology chemically and physically. Chemical crosslinking involves the use of bifunctional crosslinkers such as ECH, multifunctional carboxylic acid, and PEGDE. However, some diglycidyl ethers produce large amounts of toxic by-products under crosslinking conditions that require elimination by extensive washing, thereby affecting the hydrogel biocompatibility and environmental safety of the production process.

## 5. Types of Crosslinking Agents in the Synthesis of Cellulose-Based Hydrogels

**Figure 6:**
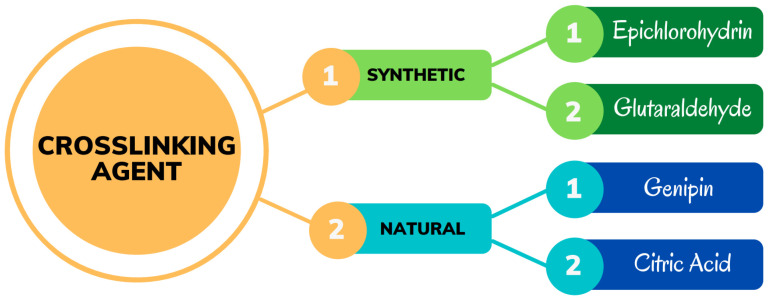
Types of crosslinking agents for cellulose-based hydrogels.

A crosslinking agent is used in hydrogel synthesis to form a three-dimensional network of hydrogels through the process of chemical crosslinking, physical linkage, ionic, and hydrogen bonding [77]. Chemical crosslinking is the formation of chemical bonds between molecular chains to form a three-dimensional network that connects molecules [78]. To synthesize the hydrogels, the crosslinking agent can be derived from natural materials and synthetic materials, as shown in Figure 6.

Depending the cellulose derivative used, several crosslinking agents can be used to form hydrogels, including ECH [6], aldehyde-based reagents [7], urea derivatives [8], and multifunctional carboxylic acids [9]. However, some reagents, such as aldehydes, are toxic in their unreacted state [10]. Even though the unreacted chemical is usually removed after crosslinking by extensive washing with distilled water, as a rule, toxic crosslinking should be avoided to maintain the biocompatibility of the final hydrogel to ensure environmentally friendly production [11].

### 5.1. Synthetic Crosslinking Agent for Cellulose-Based Hydrogels

Previous research on the development of CMCNa-based hydrogels with synthetic crosslinking agents is shown in Table 6. This table describes the synthetic crosslinking agents used in the synthesis of CMCNa-based hydrogels, which are ECH and GA.

On the basis of Table 3, Zhang and Qiao [79] successfully synthesized hydrogel with CMCNa as a polymer and ECH as a crosslinking agent. In soil, the addition of superabsorbent polymers (SAPs) can lower water evaporation and percolation. However, the repeating water absorbency (RWA) and salt tolerance of prepared SAPs do not meet the requirements for their use. This study examined the effect of valence cations (Na^+^, Ca^2+^, and Al^3+^) on the structural variations of CMCNa-based hydrogels crosslinked with ECH. The results showed that, because of the existence of more carboxyl groups, the higher addition of NaOH resulted in a higher water absorbency (WA). It was found that the sample with 5% CMCNa and 3% NaOH was a qualified hydrogel with WA of 969.0 g/g in deionized water. In the solution, the hydrophilicity and the salt resistance of the sample decreased with increasing cation valence. In the sample, the introduction of Na^+^ resulted in the replacement of H^+^ from the carboxyl group. The coordination of the Ca^2+^ and carboxyl group was tridentate bridging and bidentate chelating for the Al^3+^ and the carboxyl group. The introduction of polyvalent cations benefited the stabilization of the carboxyl group, but resulted in lower WA because of the hindered swelling ability of the CMCNa sample.

In line with previous research, Peptu et al. [80] synthesized alginate (AG)/CMCNa-based hydrogel with ECH as a crosslinking agent. It was found that high superabsorbent properties, indicated by a maximum swelling ratio of 1273%, were observed for the sample with 1:1 AG:CMC molar ratio, 6.6% polymer concentration, and 0.75 mL of ECH. This result was expected, because the SEM showed a porous structure. Despite its porous structure, another sample with a 1:1 AG:CMC molar ratio, 6.6% polymer concentration, and 3 mL of ECH had a swelling ratio of only 362%. This condition can be explained by the higher ECH concentration of the sample before, which defined a wider network of crosslinked polymers. The number of crosslinking agents affected the swelling ratio, and with few crosslinking agents, higher swelling ratios were obtained compared with the samples with a high number of crosslinking agents that showed low swelling ratios. These results indicate that the swelling ratio is dependent on both the crosslinking agent and the polymer concentration.

Khabibi et al. [81] successfully synthesized CS/CMCNa-based hydrogel with GA as a crosslinking agent. This CS/CMCNa-based hydrogel has a higher swelling ability. Additionally, the higher addition of GA causes a decrease in water adsorption. The decrease in membrane swelling is possibly due to the CS and CMC hydrophilic groups binding with GA during the crosslinking reaction. 

In line with previous research, Sritweesinsub and Charuchinda [82] synthesized AG/CMCNa-based hydrogel with GA as a crosslinking agent. The increase in the CMC-to-AG ratio on the crosslinked hydrogel with GA alone improves its swelling ratio. GA was suggested to be able to effectively crosslink at hydroxyl groups of CMC. The swelling ratio of crosslinked hydrogel with Cu^2+^ alone could be slightly improved when the AG increased due to the crosslink interaction between the carboxylate group in AG and Cu^2+^. However, when GA and Cu^2+^ were employed, it took a greater swelling time than the crosslinking agent alone (40 times). The time to reach the maximum swelling value was extended due to the formation of crosslinks between copper ion and carboxylate groups in AG similar to the formation of crosslinks between the hydroxyl group of CMC and GA. This finding shows that an increase in the AG ratio caused a decrease in the swelling ratio because of enhanced crosslink density.

### 5.2. Natural Crosslinking Agent for Cellulose-Based Hydrogels

Previous research on the development of CMCNa-based hydrogels with natural crosslinking agents is shown in Table 7. This table describes the natural crosslinking agents used in the synthesis of CMCNa-based hydrogels, which are genipin and citric acid.

Genipin (from the fruit of gardenia) is widely used as a alternative crosslinking agent to dialdehydes because of its biocompatibility. Genipin can bind polymers with biological tissues covalently, such as CS and gelatin [83]. Genipin is a natural crosslinking agent and is 10,000 times less toxic than the GA crosslinking agent, which is commonly utilized to crosslink the hydrogel with a minimum toxic effect [84].

Based on Table 7, Muhamad et al. [84] synthesized kappa-carrageenan (κC)/CMCNa hydrogel with genipin crosslinking agent. The mixture hydrogel beads of κC: CMCNa with a ratio of 90:10 swelled the fastest, followed by 80:20, 70:30, and 60:40. When the weight fraction of carrageenan increases at 90:10, the counterions in the solution (SO^3−^) also increase. The increases in the SO^3−^ ion resulted in a stronger electrostatic repulsion between the SO^3−^ groups and increased the osmotic pressure, thereby increasing the polymer swelling.

To determine the swelling response of the hydrogel to pH, a swelling test of beads was conducted in an acidic medium of pH 1.2 and a medium of pH 7.4. Most mixture ratios of beads exhibit better swelling in pH 7.4 than in pH 1.2. In the mixture ratio of 70:30 beads, the swelling degree is 109% and 100% at pH 7.4 and 1.2, respectively. The carboxylate COONa changes to COOH (acid form) at low pH. Therefore, most of the carboxymethyl groups in the form of COOH are less ionized. As the pH increases, the carboxylic groups become ionized, and the resulting repulsion in the network will cause the beads to swell. As a result, beads with a mixture ratio of 70:30 were chosen. Although beads with a mixture ratio of 80:20 and 90:10 had a better degree of swelling than beads with a mixture ratio of 70:30, they were not suitable for the formation of beads because they did not produce spherical beads. Beads with a mixture ratio of 60:40 were not chosen because their structure was not strong and could be dissolved in the pH medium.

Beads crosslinked with the highest concentration of genipin (1.5 mM) show lower swelling than 0.5 mM. A high concentration of genipin could result in a great amount of chemical crosslinking of the κC/CMCNa chains. This condition could restrict the mobility and hydration of the macromolecular chain in the beads and lead to less swelling in terms of diameter.

In recent years, citric acid has served as a non-toxic crosslinking agent for hydrogel synthesis. Demitri et al. [9] successfully synthesized CMCNa and HEC-based hydrogel as a polymer and created hydrogels with citric acid crosslinker. The SR analysis indicated that at the same citric acid concentration, the swelling of CMCNa crosslinked with 10% citric acid was higher than that of HEC. The swelling of HEC-based hydrogel was the same as that of CMCNa-based hydrogel with citric acid concentration of 20%, thereby showing that the reaction rate between citric acid and HEC was higher than the reaction rate between citric acid and CMCNa at a citric acid concentration of 20%. This condition may have occurred because HEC is less sterically obstructed than CMCNa and can react faster than the CMCNa chain.

However, CMCNa/HEC with weight ratio of 3/1 showed that at a citric acid concentration of 3.75%, an SR of 900% can be reached. These hydrogels, once swollen, were characterized by good rigidity and the ability to maintain the same form. With this finding, it can be concluded that the use of citric acid as a crosslinking agent in hydrogel synthesis is not only environmentally friendly, but also gives a higher SR. However, at citric acid concentrations lower than 1.75%, weak crosslinking between cellulose and citric acid will form, thereby producing hydrogels with an insufficient mechanical properties.

In line with previous research, Gorgieva and Kokol [55] created CMCNa/HEC hydrogel with citric acid crosslinker and found that increasing the CMCNa concentration increased the swelling capacity of the hydrogel, with an increase of 10–20% for the hydrogels made from CMCNa/HEC 3:1 compared to the hydrogels made from CMCNa/HEC 1:1. Moreover, the hydrogels made with higher HEC content were less stable because of their low crosslinking ability, as influenced by their higher substitution degree (fewer −OH groups) compared to CMCNa.

At pH 6.25 ± 0.25 (pH of distilled water), the carboxylic acid groups should be ionized (COO−), because the pKa of the carboxylic acid in the polysaccharide is 4.6. At this pH, the hydrogen bonds will be broken, thereby resulting in electrostatic repulsion between macromolecules, and water will be taken up. The hydrogel made from CMCNa/HEC 1:1 has fewer hydrogen bonds than the hydrogel from CMCNa/HEC 3:1. Moreover, the CMCNa/HEC 3:1 hydrogel crosslinked with higher (5.75%, *w*/*w*) citric acid concentration formed fewer hydrogen bonds compared with the hydrogels with 3.75% (*w*/*w*) of citric acid, and the response to changes in pH was immediate. Using 3.75% (*w*/*w*) citric acid resulted in higher and more intensive swelling in alkaline medium than in acidic medium, thereby indicating that the gels, being weakly acidic, have more ionized carboxylic groups in alkaline pH. Thus, greater electrostatic repulsion occurred between COO− groups, thereby opening the network and increasing the water uptake of the gels.

Durpekova et al. [85] studied CMCNa/HEC-based hydrogel with citric acid as a crosslinking agent and acid whey as polymeric solution. They found that the mixture CMCNa/HEC hydrogel has a higher swelling capacity than just CMCNa or HEC, with the same citric acid concentration and swelling in distilled water. The HEC-based hydrogel was less stable and showed a lower potential for absorption once citric acid was introduced. It is caused by its low crosslinking capability, which is due to a higher degree of substitution (fewer –OH groups) than that of CMCNa. CMCNa is a polyelectrolyte compound that shows ionic strength and sensitivity to pH. CMCNa increases the swelling capacity of a hydrogel as a consequence of the Gibbs–Donnan effect, thereby increasing the osmotic pressure. An increase in osmotic pressure can force water to enter the hydrogel and inhibit any rise in the ionic strength of the external solution. However, poor crosslinking efficiency has been reported when only CMCNa is utilized because of electrostatic repulsion between the charged macromolecules of polyelectrolyte chains. Thus, in hydrogels, HEC promotes the formation of intermolecular rather than intramolecular crosslinks.

As confirmed in other research, the swelling is not only dependent on the ratio of the polymer, but can also be modified by varying the amount of the crosslinking agents. When a higher concentration of citric acid was present in the polymer solution (caused by an increase in crosslinking density), lower uptake of water was observed. Moreover, hydrogels with a low concentration of citric acid were not sufficiently formed due to limited crosslinking.

Although a higher absorption capacity was observed from CMCNa/HEC (3/1) with 5.75% *w*/*w* citric acid for the samples prepared from water, the sample prepared from whey showed similar values at the citric acid concentration of 5% wt. The CMCNa/HEC hydrogel crosslinked by 5% of citric acid with 0.5% of acid whey solution (pH 4.5) showed the best swelling values. Low-protein acid whey can be used to replace the distilled water that is commonly used to synthesize hydrogels and to effectively utilize the waste product of the dairy industry. The swelling results of the whey/cellulose-based hydrogels showed high swelling capacities (1000–1700%), comparable to that of the other synthesized hydrogels from water.

Swelling media of different pH were utilized to confirm the effect of pH on the swelling capacity of cellulose/whey hydrogel. Hydrogel reached the maximum swelling capacities after it had been soaked in distilled water at pH 7.2 (1115%) and saline solution at pH 10.0 (994%). A significant decrease in swelling capacity occurred in acidic media at pH 2.5, which was caused by the protonation of the carboxyl groups. At pH values higher than the pKa of carboxylic groups (pKa 4–5), the carboxylic acid groups became deprotonated. Electrostatic repulsive forces between the negatively charged sites (COO−) can lead to enhanced water uptake capability.

The working mechanism of citric acid is that when it is heated, the carboxylic acid group in citric acid will be dehydrated, thus forming a cyclic anhydride. Then, the cyclic anhydride of citric acid crosslinks with the hydroxyl groups on the cellulose through an esterification reaction. Demitri et al. [9] explained in detail that at 60 °C, the carboxylic acid in citric acid begins to dehydrate to cyclic anhydride, and at 160 °C, the citric acid is already degraded. The thermal stability of CMCNa is observed at a temperature below 100 °C and is degraded above 100 °C. Therefore, the right temperature for the crosslinking process of CMCNa with citric acid is 80 °C.

**Figure 7:**
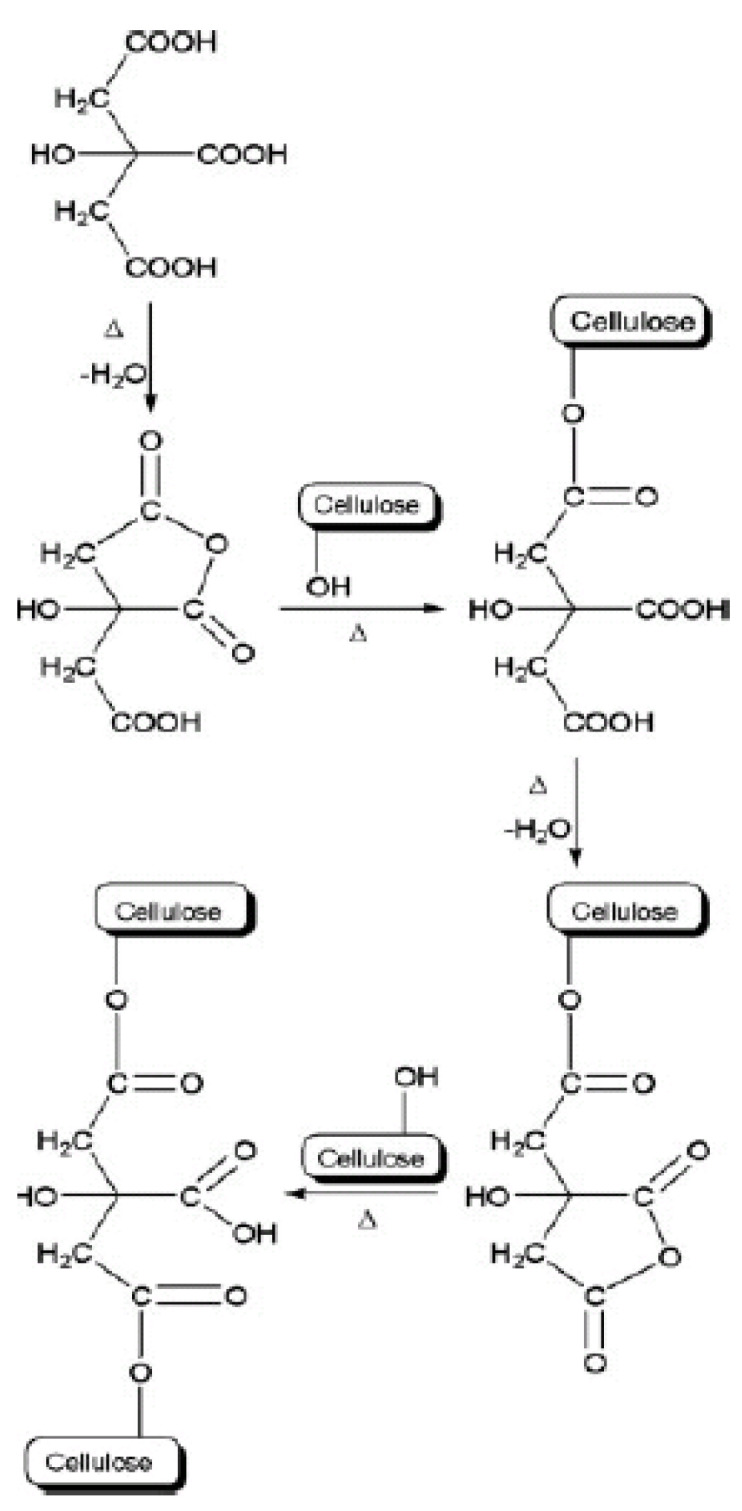
Mechanism of crosslinking of cellulose with citric acid (addapted with permission from reference [9]).

**Figure 8:**
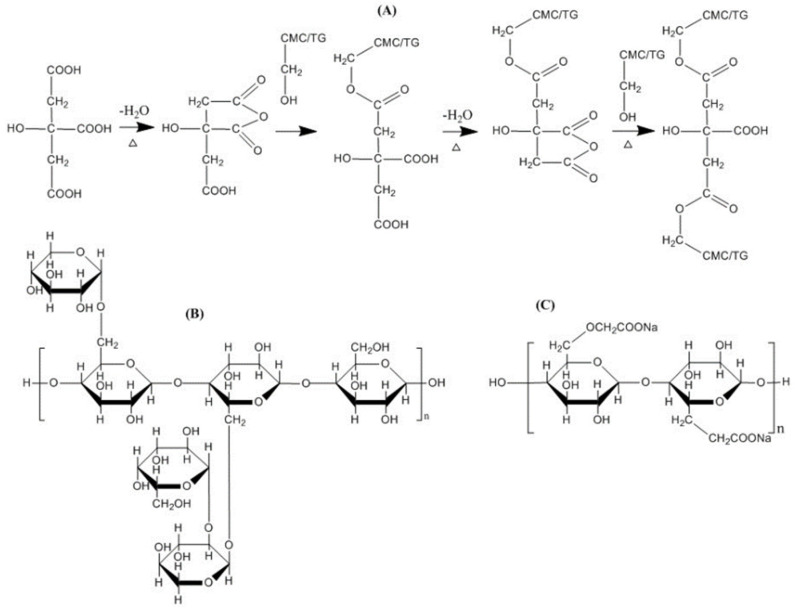
Mechanism of crosslinking of CMC/TG with citric acid [47]. Possible crosslinking reaction between citric acid, TG and CMC (**A**), structure of tamarind gum (**B**) and structure of carboxymethyl cellulose (**C**).

The following figures illustrate the crosslinking mechanism by citric acid that occurs in cellulose (Figure 7) and CMCNa (Figure 8).

**Figure 9:**
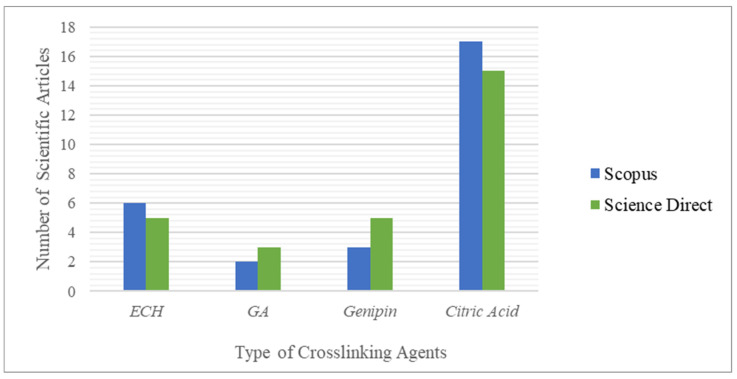
Statistics of the search results for scientific articles on Scopus and ScienceDirect 3.

According to previous research, cellulose-based hydrogels that are crosslinked with citric acid produced a hydrogel with good swelling capability, biodegradability, and safe production process [11]. However, lower water absorption was observed when higher concentrations of citric acid were present in the polymer solution. Likewise, if the concentration of citric acid is low, then crosslinking in the hydrogel is not sufficient to form a hydrogel. In addition, a trend toward the use of citric acid in hydrogel synthesis from year to year has not been observed since Demitri et al. [9] developed a citric acid crosslinked cellulose derivative-based hydrogel. This situation is evidenced by the fact that citric acid is still not frequently used as a crosslinking agent in natural hydrogels, especially in CMCNa-based hydrogels, when compared with other synthetic hydrogels that are chemically more dangerous, as shown in Figure 9. Figure 9 describes the statistics of the search results on the types of crosslinking agents.

Figure 9 was prepared using the Scopus and ScienceDirect search engines based on keywords such as “ECH crosslinked CMCNa hydrogel”, “GA crosslinked CMCNa hydrogel”, “genipin crosslinked CMCNa hydrogel” and “citric acid crosslinked CMCNa hydrogel”. The above graph shows that the use of citric acid as a crosslinking agent in the synthesis of CMCNa-based hydrogels has come a long way, and is more desirable than ECH, GA, and genipin, as evidenced by the greater number of scientific articles published on the synthesis of hydrogels based on CMCNa with citric acid as a crosslinking agent (17 and 15 articles published in Scopus and ScienceDirect, respectively, in 2011–2021). 

A greater amount of research is available because the synthesis of CMCNa-based hydrogel with citric acid as a crosslinking agent can produce higher swelling properties [9,55], offers stability on different pH swelling media [84], biodegradability, and ensures much better safety [11] than other crosslinking agents such as ECH, GA, and genipin. Another reason is that GA is more often used as a crosslinking agent for CS-based hydrogel [86,87] and genipin [88,89]. 

## 6. Conclusions

The literature review and analysis of several pieces of supporting data on the several types of polymers that can be used in the manufacture of hydrogels found that cellulose had greater potential than other polymers because it is highly biocompatible, easily accessible, and affordable. Findings prove that previous studies used CMCNa more frequently than cellulose derivatives in the manufacture of hydrogels. In addition, CMCNa hydrogels can absorb large amounts of water and expand to form superabsorbent hydrogels.

This literature review proves that many researchers have synthesized CMCNa from natural materials. The latest field of research is the synthesis of CMCNa from passion fruit peel. Thus, passion fruit peel has the potential to be used as a component in the synthesis of CMCNa-based hydrogels.

This literature review indicates that several crosslinking agents have been used in cellulose-based hydrogels or CMCNa, and citric acid is a more promising crosslinking agent than other crosslinking agents, because it comes from natural ingredients and is harmless, as evidenced by the high swelling ratio when using the appropriate crosslinking degree. Increasing the crosslinking degree of the hydrogel caused a significant reduction in the swelling ratio. However, when higher concentrations of citric acid were present in the polymer solution, lower water absorption was observed. Likewise, at a low concentration of citric acid, crosslinking in the hydrogel is not sufficient to form a hydrogel.

## Figures and Tables

**Table 1:** Research on types of hydrogels based on synthetic polymers.

Types of Hydrogels	Types of Polymers and Crosslinking Agents	Research Result	Applicability	Reference
PAAm-based hydrogel	NR-g-PAAm as a polymer and NMBA as a crosslinking agent	Hydrogel with 30% NR can swell up to 15,200% and could remove more than 90% of 50 ppm methylene blue dye. However, it has less water absorption when NR is not present.	Methylene blue dye removal	[20]
	PAAm and CA as a polymer and NMBA as a crosslinking agent	For CA concentrations more than 20 wt%, NMBA-free PAAm-CA hydrogels had a swelling capability comparable to that of formulations containing NMBA. Therefore, with CA concentrations of 20 wt% to 25 wt%, the hydrogel’s ability to swell was unaffected by the use of NMBA as a crosslinking agent.	Tissue engineering applications such as cartilage replacement	[21]
	PAAm, PAAm/CMCNa and PAAm/CMCNa/MgO as a polymer and NMBA as a crosslinking agent, APS and TEMED as initiator	The amount of swelling was increased considerably by adding CMCNa. In contrast, MgO nanoparticles had a negative effect on swelling capacity by reducing the porosity of hydrogel.	Amoxicillin or semi-synthetic antibiotic	[22]
PVA-based hydrogel	Lignin-PVA as a polymer and ECH as a crosslinking agent	The ideal swelling ratio of hydrogels made from different types of lignin can exceed 550 g/g with a lignin concentration of 5%, indicating that different forms of lignin had good compatibility with PVA. The lignin PVA hydrogel’s ability to adsorb rhodamine 6 G, crystal violet and methylene blue dyes reached 196, 169, and 179 mg/g, respectively, indicating possible uses for the removal of dye pollutants.	Dye pollutant (rhodamine 6G, crystal violet, and methylene blue) removal.	[23]
	PVA as a polymer and telechelic PVA as a crosslinking agent	The higher number of telechelic PVA as a crosslinking agent than PVA polymers can decrease the total water content (TWC). However, a lower number of telechelic PVA than PVA can increase the TWC.	Cleaning paper artworks	[24]
	PVA/CMCNa as a polymer and inebrin as a hemostatic agent	The swelling behavior of the hydrogel can be described as the water uptake of the hydrogel. The maximum swelling degree increases along with increasing concentration of CMCNa, which increases from 500% to 3200%.	Wound dressings for capillary bleeding	[25]

**Table 2:** Research on types of plant polysaccharides-based hydrogels.

Types of Hydrogels	Types of Polymers and Crosslinking Agents	Research Result	Applicability	Reference
Cellulose-based hydrogel	Sugarcane bagasse cellulose as a polymer and citric acid and ECH as a crosslinking agent	The addition of citric acid lower than 40% cannot create crosslinked networks. Thus, hydrogels cannot form and become brittle. The addition of 40% citric acid forms hydrogel with a mechanical strength comparable to that of a crosslinked hydrogel with 5% ECH. The hydrogel with citric acid has greater methylene blue dye adsorption than that with ECH.	Methylene blue dye removal	[28]
	Corncob cellulose-co -AMPS as a polymer, borax decahydrate as a crosslinking agent, and KPS as an initiator	Hydrogels made from a corncob cellulose-co-AMPS mixture have a higher swelling ratio than commercial superabsorbent polymers, which is 27,960% and 19,380%, respectively. The hydrogel showed a swelling ratio of 8330% to an application for a urine solution.	Personal hygiene	[29]
	Rice straw cellulose, CMCNa, and CMCNa/cellulose as a polymer and vs. as a crosslinking agent and GA, NMBA, and ECH for the mixture of 1:1 of cellulose: CMC.	CMCNa hydrogel, CMCNa: cellulose (1:1) (wt/wt) hydrogel, and CMCNa: cellulose (4:1) (wt/wt) hydrogel displayed high equilibrium swelling after 3 days and reached saturation on the fourth day, where they reached up to 2486%, 3477%, and 2194.7% of their weights, respectively, while cellulose hydrogel reached 7182% on the fourth day. The rate of absorption increased on the third day when the ratio of CMCNa to CMCNa/cellulose was increased, and it reached saturation on the fourth day. As opposed to 2194.7% for CMCNa: cellulose (4:1) (wt/wt) and 2486% for CMCNa alone, the equal ratio of CMCNa: cellulose (1:1) (wt/wt) can reach 3477%. VS, GA, ECH, and NMBA were used as a crosslinking agent with CMCNa:cellulose (1:1). The best absorption rate of the hydrogel was observed when GA was used as a crosslinking agent.	Removal of metals (Cu^2+^) from wastewater	[30]
Starch-based hydrogel	Cassava starch as a polymer and ECH and SEC as a crosslinking agent	The optimal swelling ratios for the ECH and SEC hydrogels were 518% and 1028.5%, respectively, with an ECH content of 5% to 10%. When the concentration of ECH is more than 5–10%, the swelling ratio is decreasing and the hydrogel is not water-soluble.	Superabsorbent	[31]
	Commercial starch as a polymer and citric acid as a crosslinking agent	The highest swelling degree of hydrogel is 8.55 at a 72 ratio of glucose units of starch and citric acid and when the pH of water is 7.	Carriers for the release of pharmaceutically active substances	[32]
	PASGC as a polymer and ECH as a crosslinking agent	The capacity of the hydrogel to swell increases with every rise in the amount of ECH up to 5 g. As the amount of ECH exceeded 5 g, the ability of the hydrogel to swell continued to decrease.	Cadmium (Cd^2+^) ion removal from aqueous solutions	[33]

**Table 3:** Research on types of animal polysaccharide-based hydrogels.

Types of Hydrogels	Types of Polymers and Crosslinking Agents	Research Result	Applicability	Reference
Chitin-based hydrogel	*Hericium erinaceus* residue carboxymethyl chitin as a polymer and ECH as a crosslinking agent	The highest DS value of 0.038 was obtained with the highest MCA concentration of 0.7 g/mL. The highest equilibrium swelling degree of 40.2 g/g was obtained with the highest DS value of 0.038. In addition, the diameter increased to a higher value from 2.40 cm to 3.51 cm with a growth rate of 46.3% for the hydrogel with the highest equilibrium swelling degree of 40.2 g/g.	Adsorption of anionic dyes	[34]
	RCNs-PEGDE as a first network and PAAm as a second network polymer and NMBA as a crosslinking agent	To overcome the mechanical weakness of chitin or RCNs, the DN strategy was efficient at adding PAAm. The swelling ratio of the DN hydrogel was higher (9.0) than that of SN hydrogel (5.4) because the DN hydrogel swelled mostly in the second network in the monomer solution.	Potential superficial soft tissue repairing materials	[35]
	Chitin/PVA as a polymer and ECH as a crosslinking agent	The equilibrium swelling ratio of 100% chitin was 52.5 and decreased gradually as the PVA content increased, thereby indicating that the gel structure became denser with PVA than the pure chitin gel.	Tissue engineering	[36]
Glycogen-based hydrogel	Glycogen-PVA and PAA as a polymer, APS initiator and Fe^3+^ as a crosslinking agent	Hydrogel with 6% of glycogen concentration showed the highest tensile stress of 1.12 MPa and fracture strain of 1420%, and obtained self-healing efficiency of 98% without any external influence	Advanced soft materials in biomedical fields	[37]
	Commercial glycogen, PAA and PAAm as a polymer, APS initiator and iron (III) as a crosslinking agent	Tensile stress and strain with 2% glycogen are 0.52 MPa and 1130%, respectively. The values continued to increase until 6% glycogen, at which point the highest tensile stress of 1.12 MPa and fracture strain of 1420% values were reached. The toughness increased to 10.71 MJ m^−3^ until 6% glycogen and decreased at 8% glycogen concentration.	Wearable strain-sensor for flexible e-skin	[38]
	Glycogen, NIPAm as a polymer, and EGDMA as a crosslinking agent	A steady decrease in the elastic modulus (G’) and loss modulus (G’’) was observed and after a certain shear stress, both modulus declined rapidly, which indicates the breakup of hydrogel. The equilibrium swelling ratio in acidic medium (pH 1.2) was lower than that in basic medium (pH 7.4). The swelling ratio at 37 °C was lower than at 25 °C.	Colon-targeted delivery of ornidazole and 5-amino salicylic acid	[39]

**Table 4:** Research on types of bacteria polysaccharide-based hydrogels.

Types of Hydrogels	Types of Polymers and Crosslinking Agents	Research Result	Applicability	Reference
Bacteria-based hydrogel	BC and CMCNa as a polymer	Both BC and BC/CMCNa hydrogels had relative cell viability higher than 80%, indicating relatively good biocompatibility. At 563 °C, BC completely decomposed. At a higher temperature (633 °C), BC/CMCNa experienced a weight loss of 100%, indicating that the presence of CMCNa increases the thermal stability.	Non-invasive semi-quantitative sensors for on-skin health monitoring	[40]
	BC and gelatin as a polymer and GA as a crosslinking agent	In water, the swelling ratio of the hydrogel network was 400–600%.	Drug-delivery systems	[41]
	BC and chitosan (CS) as a polymer and GA as a crosslinking agent	The highest expansion ratio of hydrogel with high CS content (ratio BC:CS of 20:80), which was impregnated with silver sulfadiazine (SSd) in an acidic medium. The swelling ratio increased in line with the BC content in alkaline media.	Biomedical fields	[42]

**Table 5:** Research on types of cellulose derivative-based hydrogels.

Types of Hydrogels	Types of Polymers and Crosslinking Agents	Research Result	Applicability	Reference
MC-based hydrogel	MC as a polymer and citric acid as a crosslinking agent	The equilibrium swelling ratio of hydrogel was different from that of crosslinked MC hydrogel with control MCs, with average expansion values from 800% for MCs with 5% citric acid to 3000% MCs with 3% citric acid. MC with 1% citric acid did not show a significant difference in the swelling equilibrium compared with MC control.	Cell sheet engineering	[48]
	MC as a polymer, citric acid as a crosslinking agent and sorbitol as a plasticizer	The addition of 0.25% plasticizer affected the barrier properties of hydrogel unlike hydrogel without plasticizer. Adding 5% citric acid to MC hydrogel plasticized with 0.25% sorbitol was possibly able to improve the barrier properties and decrease the affinity for water.	Controlled release agents or in the food industry	[54]
HEC-based hydrogel	HEC as a polymer, citric acid as a crosslinking agent and WO_3_ as a support material	The gel fraction of hydrogel without WO_3_ was 59.7% and increased to 65.9% after the addition of 0.02% WO_3_. The highest swelling of hydrogel was achieved for hydrogel without WO_3_ and with 0.02% WO_3_.	Wound dressing material	[49]
	HEC-g-PNaS/medical stone as a polymer and NMBA as a crosslinking agent	Unlike the MS-free sample, the addition of 10% medical stone increased the swelling capacity by 400%, and the initial expansion rate constant increased by 7.48 times. Adding medical stone increased the initial swelling rate but decreased with increasing ion strength.	Petroleum-based synthetic absorbents	[58]
HPMC-based hydrogel	HPMC as a polymer and BCP	The incorporation of HAp and TCP nanoparticles on BCP in HPMC aqueous solution increased the viscosity of injection scaffold but decreased the gelation temperature.	Support of metal dental implants	[50]
	HPMC and pectin as a polymer, AA as a monomer, and NMBA as a crosslinking agent	With a higher pectin quantity, the swelling percentage increased from 79.58% to 92.62% at pH 7.4. Increasing the amount of HPMC from 0.5 g to 1.5 g affected the percentage of swelling so that the swelling increases from 76.68% to 95.89% at pH 7.4.	Controlled-delivery drug for dementia	[61]
HPC-based hydrogel	HPC as a polymer and MoS_2_ as a crosslinking agent	When compared with HPC hydrogels at 25 °C, the MoS_2_-HPC/HPC hydrogels had a smaller expansion ratio as a result of the MoS_2_-HPC crosslinking action.	Methylene blue dye removal	[3]
	HPC as a polymer, ECH as a crosslinking agent and ammonium as a co-crosslinking agent	The cationic HPC hydrogel showed an excellent ability to adsorb anionic dye (dye orange (II)), and the maximum adsorption capacity at room temperature was 2478 (g/kg) at pH 3.96.	Adsorb anionic dye	[65]
CMCNa-based hydrogel	CMCNa as a polymer and ECH as a crosslinking agent	The highest WRV was 725 g distilled water/g gel and 118 g saline-water/g gel, with a composition of 3% of CMCNa and 4% of ECH.	High-value hygiene	[51]
	CMCNa and HEC as a polymer, divinyl sulfone as a crosslinking agent	As the temperature increased, the weight loss of CMCNa and crosslinked CMCNa/HEC hydrogel indicated the loss of moisture. The temperature of decomposition (TD) was 285.5 °C (weight loss: 68.2%) of CMCNa and 276.6 °C (weight loss: 56.8%) of crosslinked CMCNa/HEC (5/1).	Water absorption	[66]

**Table 6:** Research on the types of synthetic crosslinking agent for cellulose-based hydrogels.

Types of Hydrogels	Research Result	Applicability	Reference
ECH crosslinked CMCNa hydrogel	The effect of valence cations (Na^+^, Ca^2+^, and Al^3+^) on structural variations of CMCNa-based hydrogels crosslinked with ECH. The existence of more carboxyl groups and the higher addition of NaOH resulted in higher WA. The sample with 5% CMCNa and 3% NaOH was a qualified hydrogel with WA of 969.0 g/g in deionized water.	Water absorption	[79]
ECH crosslinked AG/CMCNa hydrogel	The high superabsorbent property indicated by the highest swelling ratio of 1273% was observed for the sample with 1:1 of AG:CMC molar ratio, 6.6% polymer concentration, and 0.75 mL of ECH. Despite its porous structure, another sample with a 1:1 AG:CMC molar ratio, 6.6% polymer concentration and 3 mL of ECH had a swelling ratio of only 362%.	Controlled drug delivery systems	[80]
GA crosslinked CS/CMCNa hydrogel	Hydrogel with the blending membrane of CS and CMC had a higher water absorption than hydrogel with CS. The greater addition of GA could effect a decrease in water adsorption.	Hemodialysis membranes	[81]
GA crosslinked AG/CMCNa hydrogel	The increase in the CMC-to-AG ratio on the crosslinked hydrogel with GA alone improved its swelling ratio. The swelling ratio of crosslinked hydrogel with Cu^2+^ alone could be slightly improved with increasing AG. However, the use of GA and Cu^2+^ resulted in a greater swelling time than the crosslinking agent alone. An increase in the AG ratio caused a decrease in the swelling ratio.	Water absorption	[82]

**Table 7:** Research on the types of natural crosslinking agent for cellulose-based hydrogels.

Types of Hydrogels	Research Result	Applicability	Reference
Genipin crosslinked kappa-carrageenan (𝜅C)/CMCNa hydrogel	Swelling ratio versus time for pH of 7.4 and 1.2. The hydrogel beads of 𝜅C: CMCNa with a 90:10 ratio swelled the fastest. Most mixture ratios of beads showed a higher swelling in pH 7.4 medium than in acidic medium (pH 1.2). In a mixture ratio of 70:30 beads, the swelling degree of bead reached 109% and 100% in pH 7.4 medium and acidic medium, respectively. The swelling of beads crosslinked with 0.5 mM genipin reached 95.24% and 100% in pH 1.2 and 7.4 medium, respectively. In contrast, beads crosslinked with the highest concentration of genipin (1.5 mM) showed the lowest percentage increase in diameter, being approximately 76.2% in acidic medium of pH 1.2 and 85.71% in the medium of pH 7.4.	Beta-carotene release	[84]
Citric acid crosslinked CMCNa, HEC, and CMCNa/HEC hydrogel	At the same citric acid concentration, the swelling of CMCNa crosslinked with 10% of citric acid was higher than that of HEC. When 20% citric acid was added, the swelling was similar for CMCNa and HEC. The highest swelling ratio was obtained with the concentration of citric acid (1.75, 2.75%, and 3.75% by weight of the mixtures). CMCNa/HEC with a weight ratio 3/1 showed that with 3.75% citric acid, the highest swelling ratio (SR) of 900% was obtained.	Superabsorbents in agriculture	[9]
Citric acid crosslinked CMCNa, HEC, and CMCNa/HEC hydrogel	The increase in the CMCNa concentration on hydrogels from CMCNa/HEC 3:1 increased the hydrogel swelling capacity by about 10–20% on average compared with the hydrogels made from CMCNa/HEC 1:1. Using 3.75% (*w*/*w*) citric acid on CMCNa/HEC 3:1 increased the swelling degree more intensively in alkaline medium than in acidic medium.	Functional finishing of cotton knitwear	[55]
Citric acid crosslinked CMCNa, HEC and CMCNa/HEC hydrogel	The CMCNa/HEC hydrogel showed a higher swelling capacity than either CMCNa or HEC hydrogel alone, with the same citric acid concentration and swelling in distilled water. When a higher concentration of citric acid was added to the polymeric solution (caused by an increase in crosslinking density), a lower uptake of water was observed. Although a higher absorption capacity was observed from CMCNa/HEC (3/1) with 5.75% *w*/*w* CA prepared from water, the sample with whey exhibited similar values at the CA of 5% wt. The CMCNa/HEC hydrogel crosslinked by 5% CA with 0.5% acid whey solution (pH 4.5) showed the highest swelling values. Hydrogels can reach their maximum swelling capacities after immersion in distilled water at pH 7.2 (1115%) and saline solution at pH 10.0 (994%), respectively. Conversely, a considerable decrease in swelling capacity occurred in the acidic medium at pH 2.5.	Agricultural material for replacing synthetic acrylic-based absorbents	[85]

## Data Availability

Not applicable.
